# Cell State and Cell Type: Deconvoluting Circulating Tumor Cell Populations in Liquid Biopsies by Multi-Omics

**DOI:** 10.3390/cancers15153949

**Published:** 2023-08-03

**Authors:** Lisa Welter, Serena Zheng, Sonia Maryam Setayesh, Michael Morikado, Arushi Agrawal, Rafael Nevarez, Amin Naghdloo, Milind Pore, Nikki Higa, Anand Kolatkar, Jana-Aletta Thiele, Priyanka Sharma, Halle C. F. Moore, Jennifer K. Richer, Anthony Elias, Kenneth J. Pienta, Amado J. Zurita, Mitchell E. Gross, Stephanie N. Shishido, James Hicks, Carmen Ruiz Velasco, Peter Kuhn

**Affiliations:** 1 Convergent Science Institute in Cancer, Michelson Center for Convergent Bioscience, University of Southern California, Los Angeles, CA 90089, USA; l.welter@gmx.de (L.W.); serenazzheng@gmail.com (S.Z.); msetayes@usc.edu (S.M.S.); morikado@usc.edu (M.M.); arushiag@usc.edu (A.A.); rnevarez@usc.edu (R.N.); naghdloo@usc.edu (A.N.); pore.milind@gmail.com (M.P.); nhiga@usc.edu (N.H.); kolatkar@usc.edu (A.K.); j.a.thiele@gmx.de (J.-A.T.); sshishid@usc.edu (S.N.S.); carmenrv_22@hotmail.com (C.R.V.); 2 Department of Biological Sciences, Dornsife College of Letters, Arts, and Sciences, University of Southern California, Los Angeles, CA 90089, USA; 3 Department of Aerospace and Mechanical Engineering, Viterbi School of Engineering, University of Southern California, Los Angeles, CA 90089, USA; 4 Department of Medicine, Keck School of Medicine, University of Southern California, Los Angeles, CA 90033, USA; 5 University of Kansas Medical Center, Westwood, KS 66205, USA; psharma2@kumc.edu; 6 Cleveland Clinic Taussig Cancer Institute, Cleveland, OH 44195, USA; mooreh1@ccf.org; 7 University of Colorado Cancer Center, University of Colorado Anschutz Medical Campus, Aurora, CO 80045, USA; jennifer.richer@cuanschutz.edu (J.K.R.); anthony.elias@cuanschutz.edu (A.E.); 8 The Cancer Ecology Center, Brady Urological Institute, Johns Hopkins School of Medicine, Baltimore, MD 21287, USA; kpienta1@jhmi.edu; 9 Department of Genitourinary Medical Oncology, MD Anderson, Houston, TX 77230, USA; azurita@mdanderson.org; 10 Lawrence J. Ellison Institute for Transformative Medicine, Los Angeles, CA 90064, USA; mgross@eitm.org; 11 Department of Biomedical Engineering, Viterbi School of Engineering, University of Southern California, Los Angeles, CA 90089, USA; 12 Catherine & Joseph Aresty Department of Urology, Institute of Urology, Keck School of Medicine, University of Southern California, Los Angeles, CA 90033, USA; 13 Norris Comprehensive Cancer Center, Keck School of Medicine, University of Southern California, Los Angeles, CA 90033, USA

**Keywords:** liquid biopsy, circulating tumor cells, circulating endothelial cells, epithelial–mesenchymal transition, breast cancer, prostate cancer

## Abstract

**Simple Summary:**

In addition to millions of normal red and white blood cells, the blood of cancer patients contains a variety of very rare cell types associated with the tumor. A simple blood draw or ‘liquid biopsy’ can, thus, provide important information about the state of the disease no matter where it exists in the body. In this paper, we describe a method for imaging all rare cells in a blood draw and the means to define each cell type using a combination of genomic, proteomic, and morphological measurements to distinguish tumor cells from mesenchymal and endothelial cells that are present in the tumor microenvironment. We compare the results of this assay among multiple prostate and breast cancer patients and show that this cellular profiling method is valid across patients and cancer types and may provide important biomarkers for assessing disease in real time.

**Abstract:**

Bi-directional crosstalk between the tumor and the tumor microenvironment (TME) has been shown to increase the rate of tumor evolution and to play a key role in neoplastic progression, therapeutic resistance, and a patient’s overall survival. Here, we set out to use a comprehensive liquid-biopsy analysis to study cancer and specific TME cells in circulation and their association with disease status. Cytokeratin+, CD45- circulating rare cells (CRCs) from nine breast and four prostate cancer patients were characterized through morphometrics, single-cell copy number analysis, and targeted multiplexed proteomics to delineate cancer cell lineage from other rare cells originating in the TME. We show that we can detect epithelial circulating tumor cells (EPI.CTC), CTCs undergoing epithelial-to-mesenchymal transition (EMT.CTC) and circulating endothelial cells (CECs) using a universal rare event detection platform (HDSCA). Longitudinal analysis of an index patient finds that CTCs are present at the time of disease progression, while CECs are predominately present at the time of stable disease. In a small cohort of prostate and breast cancer patients, we find high inter-patient and temporal intra-patient variability in the expression of tissue specific markers such as ER, HER2, AR, PSA and PSMA and EpCAM. Our study stresses the importance of the multi-omic characterization of circulating rare cells in patients with breast and prostate carcinomas, specifically highlighting overlapping and cell type defining proteo-genomic characteristics of CTCs and CECs.

## 1. Introduction

Carcinomas are composed of a heterogeneous collection of cells with distinct genomic, proteomic, and morphometric properties. They are surrounded by a multitude of non-cancerous cell types forming a diverse microenvironment which supports the tumor’s growth [1]. Emerging evidence highlights the bi-directional crosstalk between tumor cells and their surrounding microenvironment, with the ability to induce changes in the cellular state, such as the epithelial–mesenchymal transition (EMT) in tumor cells, stressing the importance of studying cancers in the context of their TME [2,3,4,5].

Liquid biopsies provide a unique opportunity to assess tumor-derived analytes in the circulatory system throughout a patient’s course of disease. While various studies have provided insight into the benefit of circulating tumor cell (CTC) enumeration and molecular characterization [6,7], increasing evidence underlines the need of going beyond the current enrichment, identification, and characterization of CTCs to represent the wider spectrum of tumor-derived analytes [8].

The primary challenge in liquid-biopsy research is the rarity of tumor-related analytes with often unknown or insufficient information to characterize these cells in the context of the blood microenvironment. As cells may change their cellular state to accommodate different microenvironments (e.g., EMT, vascular mimicry [9] and tumor dormancy), assessing CTCs in the blood from patients with carcinomas requires the use of a spectrum of markers. Given the plasticity of both tumor cells and the TME, it is essential to use a platform that allows for the characterization of disease-associated cells by cell type and state in the context of the normal cells.

EMT, a well-known cell state change in tumor cells and one of the proposed mechanisms for promoting CTC formation, has been associated with resistance to chemotherapy and a worse prognosis [10,11,12,13]. While EMT has been viewed initially as a binary process, recent research indicates that EMT is rather a gradual transformation from an epithelial to mesenchymal state, including a partial EMT (pEMT) state, where cancer cells exhibit both epithelial and mesenchymal markers [14,15]. Under pathological conditions, cells in adult tissues rarely complete the entire EMT program, indicating that pEMT represents the norm rather than the exception [15].

Angiogenesis, one of the hallmarks of cancer, describes the growth of new blood vessels in response to stimuli from the TME to supply growing tumors with oxygen and nutrients to sustain tumor growth [16]. Consequently, the tumor endothelium has been an important target of anti-cancer therapies [17]. Circulating endothelial cells (CECs), while mainly studied in the context of vascular damage and dysfunction in cardiovascular diseases [18,19], have been proposed as a biomarker in cancer patients [20,21,22,23]. As of today, CECs have been specifically studied in patients with breast, colorectal and small-cell lung cancer (SCLC) receiving anti-angiogenic therapies, and increases in CEC counts have been associated with prolonged progression-free survival [24,25,26,27]; yet, their predictive value remains controversial [21,22]. In addition, little is known about the significance and frequency of CECs in patients with carcinomas not treated with anti-angiogenic drugs.

In 2014, Dago et al. described heterogenous CD45-/CK+ positive CTC populations at four sequential time points during the treatment of a metastatic castrate-resistant prostate cancer patient (mCRPC). They reported that at least two cell types were present in each of the four sequential blood draws with distinct cell types dominating at different timepoints. Single cell copy number profiling showed that one population carried the genomic alterations characteristic of the bulk tumor profile. In contrast, the second population was genomically neutral and morphologically distinct, indicating that these cells were not part of the cancer lineage, but were likely cells shed from the TME. However, at that time, the specific cell type remained unknown.

Here, we reveal in a longitudinal analysis of archived slides from that same index patient that CTCs and CECs, as well as cell state changes of the former, are associated with different treatment responses. To determine if these observations exist in other cases, we explored the liquid biopsy of a small cohort of breast and prostate cancer patients, in which we demonstrate the detection and characterization of rare cells that are epithelial CTCs (EPI.CTC), CTCs undergoing partial EMT (pEMT.CTC) and cells consistent with circulating endothelial cells. Together, this research demonstrates the complexity of cell populations in cancer patients and emphasizes the biopsy methods capable of deconvoluting and quantifying multiple types of rare circulating cell types and cell states to achieve the full benefit of LBx in precision oncology.

## 2. Materials and Methods

### 2.1. Patients and Samples

Patients were selected from multiple studies based on the presence of rare cells. Cancer subtype, stage and IRBs are summarized in Table 1, Table 2 and Table 3. BC samples 2, 3, 5, and 6 were collected as part of the SWOG 1416 trial (NCT02595905). The BC7 sample were collected as part of the SWOG 1222 trial (NCT02137837).

### 2.2. Blood Sample Collection and Processing

**Figure 1:**
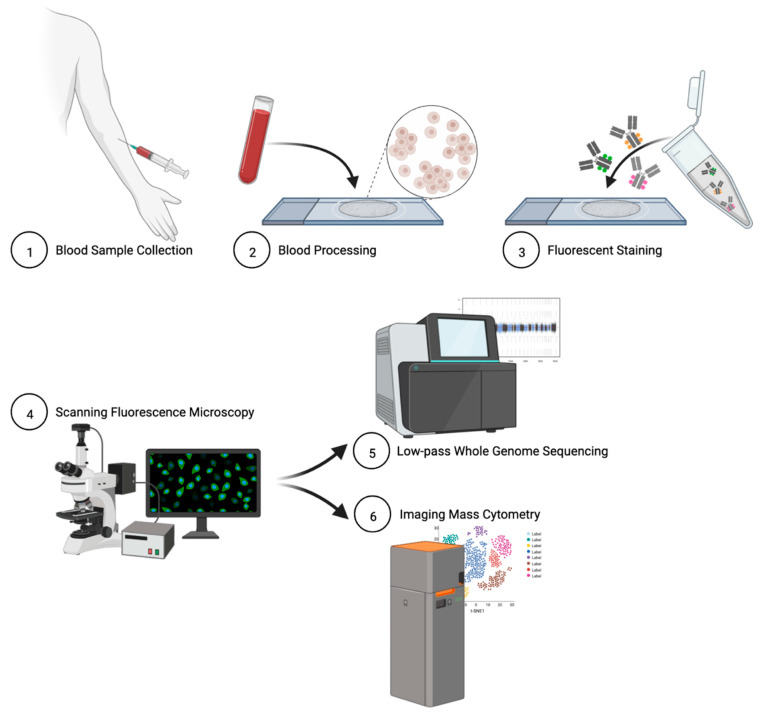
Schematic overview of HDSCA platform.

Peripheral blood samples were collected in Streck cell-free DNA (cfDNA) blood-collection tubes and shipped to the central laboratory. Blood sample processing and slide preparation for detection have been previously described [2,28]. In short, blood samples underwent erythrocyte lysis in isotonic ammonium chloride solution and nucleated cells were plated onto custom adhesive glass slides (Marienfeld) as a monolayer of approximately 3 × 10^6^ cells. Slides were incubated for 40 min at 37 °C, treated with 7% BSA, and stored in a biorepository at −80 °C for later analysis. Figure 1 summarizes the High-Definition Single Cell Analysis (HDSCA) blood-processing platform.

### 2.3. Cell Culture and Contrived Sample Generation

HPAEC (cat #PCS-100-022) and HUVEC (cat#PCS-100-013) cells were purchased from ATCC and cultured in Vascular Cell Basal Medium (cat#PCS-100-030) supplemented with VEGF (Endothelial Cell Growth Kit-VEGF, cat#PCS-100-041). Normal blood donor (NBD) peripheral blood samples were collected in Streck cfDNA blood collection tubes at the Scripps NBD Service and processed as previously published [28,29]. Cell-line cells were spiked into the NBD sample at 100 cell/mL (HUVEC) and 430 cells/mL (HPAECs).

### 2.4. Immunofluorescent Staining of Patient Slides

Slides underwent fluorescent staining as described previously [2,28]. In short, cells were incubated with an antibody mix consisting of conjugated mouse anti-human CD45 Alexa Fluor^®^ 647 (clone: F10-89-4, MCA87A647, AbD Serotec, Raleigh, NC, USA), a cocktail of mouse IgG1/Ig2a anti-human cytokeratins (CK) 1, 4, 5, 6, 8, 10, 13, 18, and 19 (clones: C-11, PCK-26, CY-90, KS-1A3, M20, A53-B/A2, C2562, Sigma, St. Louis, MO, USA), mouse IgG1 anti-human CK 19 (clone: RCK108, GA61561-2, Dako, Carpinteria, CA, USA), and rabbit IgG anti-human vimentin (Vim) Alexa Fluor^®^ 488 conjugated (clone: D21H3, 9854BC, Cell Signaling, Danvers, MA, USA) for 2 h. Slides were then incubated with Alexa Fluor^®^ 555 goat anti-mouse IgG1 antibody (A21127, Invitrogen, Carlsbad, CA, USA) and counter-stained with 4′,6-diamidino-2-phenylindole (DAPI; D1306, Thermo Fisher Scientific, Waltham, MA, USA) for 1 h. Slides were finally mounted with a glycerol-based aqueous mounting media to enable future coverslip removal for downstream genomic and proteomic analyses without disrupting cell integrity.

### 2.5. Rare Cell Identification and Characterization

Slides were imaged with an automated high-throughput microscope with a 10x optical lens, and candidate cells were identified based on their marker expression (DAPI+/CK+/CD45- with variable Vim expression) and morphological features, as previously described [28]. All identified candidate cells were presented to a trained analyst for verification. CK positivity was defined as six standard deviations over the mean (SDOM) signal intensity relative to the surrounding leukocytes (negative control for CK) for the initial candidate-cell selection. Vim expression was scored as positive or negative in CK+ candidate cells based on the image intensity within the cell mask and expression was reported as raw fluorescent intensity (RFI).

### 2.6. Single-Cell Next-Generation Sequencing and Bioinformatic Analysis

Candidate cells from patients (BC 1–6 and PC 1–2) were isolated for whole-genome amplification as previously described [30,31]. Briefly, single cells were isolated from the slide using a robotic fluid micromanipulation system (Eppendorf, Hamburg, Germany) and were deposited into individual PCR tubes for whole-genome amplification. Single cells underwent whole-genome amplification (WGA) using the WGA4 Genomeplex Single Cell Whole-Genome Amplification Kit (Sigma-Aldrich, St. Louis, MO, USA) followed by purification with the QIAquick PCR Purification Kit (QIAGEN, Germantown, MD, USA). DNA concentration was measured using the Qubit Fluorometer system (Thermo Fisher Scientific, Waltham, MA, USA). Single-cell libraries were constructed using the NEBNext^®^ Ultra™ II FS DNA Library Prep Kit with NEBNext^®^ Multiplex Oligos (New England Biolabs, Ipswich, MA, USA) and sequenced at the USC Dornsife Sequencing Core to generate ~500,000 mapped reads per sample (minimum 250,000).

To create copy number alteration (CNA) profiles, samples underwent bioinformatic analysis as previously published [32]. In summary, reads were deconvoluted based on sample barcodes and PCR duplicates were removed. Next, binned ratios were normalized based on the guanine–cytosine (GC) content per bin and mapped to 5000 bins across the human genome (hg19, Genome Reference Consortium GRCh37, UCSC Genome Browser database). The CBS algorithm was used to segment the read count data which was used to generate copy-number profiles [33]. Gains were defined as >1.25 and losses as 0.75 over the median. Heatmaps were generated using the in R using the heatmap.2 function in the ggplots package. Clonality was defined as two or more cells with shared CN breakpoints. Additionally, if only one altered cell was detected as in the case of BC5, cells were considered cancerous and therein referred to as clonal cells, if their CNAs conformed with those commonly found in the respective cancer type [34,35].

### 2.7. Single-Cell Targeted Proteomics and Data Analysis

Following rare-cell detection as described above, patient slides (BC 2, BC 7–9 and PC 1–4) were washed and re-stained with metal-conjugated antibodies (Table 4) as previously described [36]. A DNA intercalator and a membrane stain were used as counterstains. Slides were dried overnight prior to laser ablation with the HyperionTM Imaging System using 0.4 mm × 0.4 mm regions of interest (ROI) centered around each cell of interest (COI). PC1 and PC2 had multiple slides analyzed with separate antibody panels.

After laser ablation and ion counting, cells were segmented using ilastik’s random forest classifier ([37] v1.3.3) (ilastik feature settings: 0.3, 0.7, and 1.0 sigma for color/intensity, edge, and texture) and ilastik’s probability masks were used in CellProfiler ([38] v2.2.0) to create single-cell masks for all samples. COIs identified by a trained analyst during the rare-cell detection step were relocated based on the cell plating pattern and confirmed by their protein expression consistent with the prior IF data. Background ion counts, defined here as negative mask space, were subtracted from ion counts within masked areas. COI mask-specific data was extracted together with data from ~150 WBCs per cancer type, and data was normalized to generate z-scores. Regions of interest (ROIs) were visualized by histocat++ [39].

### 2.8. Data Analysis and Visualization

Data were visualized with GraphPad Prism (version 8.0.0 for Windows, GraphPad Prism Software, version 9.0.2, San Diego, CA, USA) and RStudio (RStudio version 1.2.1335, Boston, MA, USA). Illustrations were designed in Biorender and Microsoft PowerPoint. Statistical analysis was performed with GraphPad Prism.

## 3. Results

### 3.1. Characterization of Circulating Rare Cells Appearing at Time Points of Progression and Stable Disease of a Metastatic Prostate Cancer Patient

In order to further investigate the heterogenous CD45-/CK+ positive CTC populations observed at four sequential time points during the treatment of our metastatic castrate-resistant prostate cancer patient (mCRPC) described in our previous report [30], we applied an additional phenotypic marker (vimentin), along with additional molecular approaches, and improved analytics, to previously unstained slides from that index patient. In the original report, at least two cell types were reported present at each timepoint, with distinct cell types dominating at different timepoints. Three draws were taken at the times of disease progression (draws 1, 2 and 4), which were dominated by genomically altered cells that were amplified for the androgen receptor (AR) and exhibited the genomic copy number pattern of the metastatic tissue [30]. Morphologically, these altered cells were AR+ by immunofluorescence and round. In contrast, at the time of stable disease (draw 3), the majority of cells were copy number neutral, AR- and elongated [30].

**Figure 2:**
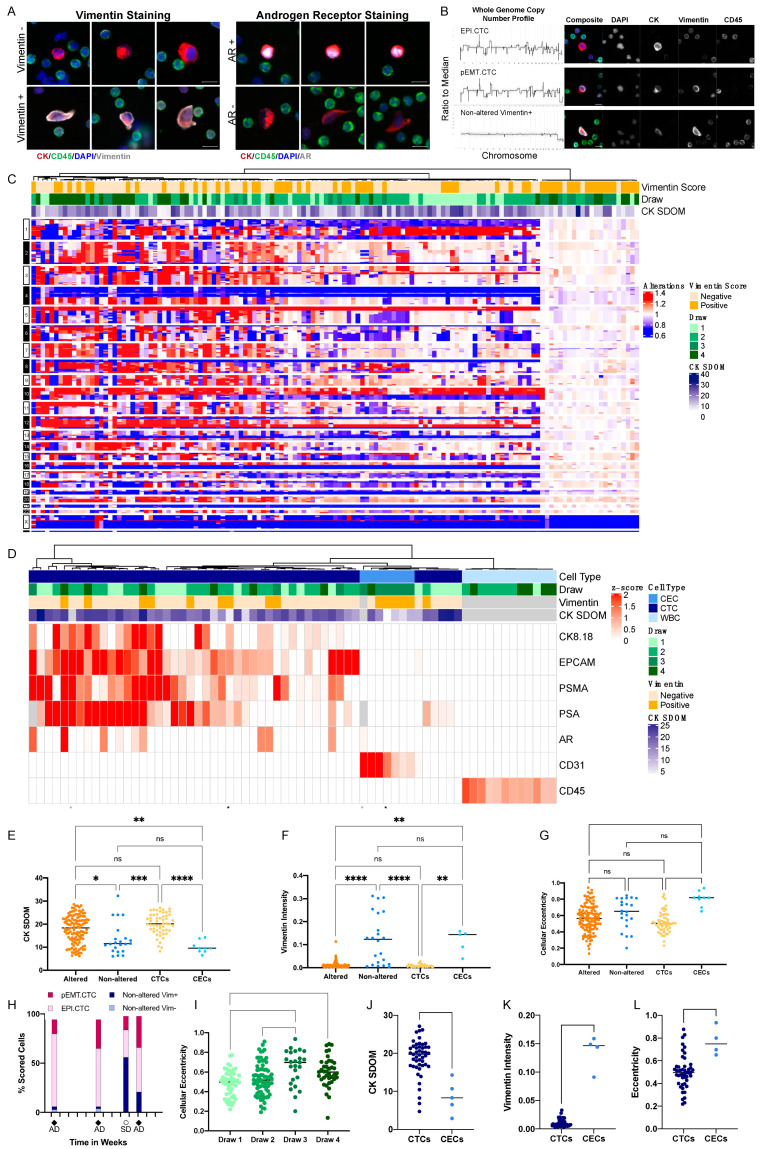
Longitudinal assessment of circulating rare cells in a patient with metastatic prostate cancer (PC1). (**A**) Representative cells organized by their Vim and AR expression. (**B**) CNA profiles together with IF images of the representative copy number of altered Vim− EPI.CTCs, copy number of altered Vim+ pEMT.CTCs and non-altered Vim+ CECs. (**C**) Copy number alterations of rare cells across four blood draws. (**D**) Multiplex protein expression of rare cells and CD45+ white blood cells across four blood draws. Cells with no Vim score available are color-coded grey in the top heatmap annotation. (**E**) CK intensity measured as standard deviation over the mean (SDOM). (**F**) Vim intensity and (**G**) cellular eccentricity from immunofluorescence image analysis of all cells with downstream NGS or multiplex proteomic data. Cells were scored as either clonal or non-altered based on the CNV profiles or as CTC or CEC based on the results from targeted proteomics. The Kruskal–Wallis test with Dunn’s correction for multiple comparisons was used to test for differences between each group. *p*-value annotations: 0.1234 (ns), 0.0332 (*), 0.0021 (**), 0.0002 (***), <0.00001 (****). (**H**) Longitudinal assessments across four blood draws. Cells were grouped by EPI.CTC (clonal, Vim−), pEMT.CTC (clonal, Vim+), and CEC (genomically non-altered, morphologically consistent with endothelial cell). Tick marks on the *x*-axis were set to 4-week intervals. Percentages of cell types might differ slightly between the total cells found per draw by the imaging microscope and those sequenced, as not all cells can be sequenced, and the sequenced cell population is, hence, a subset of the total cells detected. (AD = Active Disease; SD = Stable Disease). (**I**) Cellular eccentricity of CTCs and CECs per draw of cells that underwent IMC. (**J**) CK SDOM, (**K**) Vim intensity and (**L**) cellular eccentricity of CTCs and CECs across draws analyzed by multiplex proteomics. Scale bars are 10 μm.

Consistent with our published results, we first characterized the two subpopulations of CD45-/CK+ rare cells, according to morphology and CK expression. The round, CKbright cell population was observed at a high abundance (~90% of the total rare cell population) in the progression draws (draws 1, 2, 4), but at a lower abundance (~25% of the total rare cell population) in the draw collected during stable disease (draw 3). The elongated, CKdim cells were predominantly present (~75% of the total rare cell population) at the time of stable disease (draw 3), but at low abundance (<10% of the total rare cell population) in the draws taken at time of progression (draw 1, 2, 4). The CKdim cells match the morphometric features of non-altered, elongated, AR- cells as found in the study of Dago et al. (Figure 2A), while the round CKbright cells presented with a morphology similar to the AR+ population in the study of Dago et al.

Single cell genomic analysis found two distinct genomic patterns, one with complex copy number alterations shared across all altered cells and one lacking distinct copy number alterations (Figure 2B,C). Clonally altered cells had, on average, a significantly higher CK expression (Figure 2E) compared to the non-altered cells. The clonally altered cell population had complex copy number profiles consistent with the reported tumor profile from the study of Dago et al. In addition, we found that the majority (19/22) of copy number-neutral cells were Vim+ (Figure 2C,F). In contrast, although 60% the clonal cells were Vim- (RFI range: 0.0025–0.0221), ~40% of the clonal cells exhibited varying levels of vimentin staining (RFI range: 0.0032–0.0522), suggesting a partial EMT shift towards a mesenchymal cell state and defining these cells as partial EMT CTCs (pEMT.CTC) (Figure 2C,F).

To further characterize the rare cell types and cell states across the blood draws of the index case, we performed targeted proteomics using the HyperionTM+ imaging mass cytometry system. Using the panel of cell differentiation markers (Table 4), we identified three major proteomic profiles among the circulating rare cells which correspond to unique cell types (Figure 2D). The largest subset of cells was characterized by the expression of prostate cancer-specific proteins, PSA and PSMA, as well as EpCAM and AR defining those as prostate-derived epithelial CTCs, but individual cells differed in their expression of the mesenchymal marker vimentin (EPI.CTCs and pEMT.CTCs). Approximately 40% of the cells expressing prostate-specific markers were Vim+ by IF staining, supporting the interpretation that a substantial subpopulation of CTCs might be undergoing a transition to a mesenchymal state (EMT). While it is impossible to perform genomic and proteomic analysis on the same cell, the proportions are consistent with our interpretation of the CNV analysis. The second subset was negative for PSMA and PSA in the proteomic analysis but uniquely positive for the endothelial protein CD31. The majority of these cells were also Vim+ and elongated by immunofluorescence image analysis, consistent with the interpretation that these are circulating endothelial cells (CECs; Figure 2D,F,G). The hypothesized CECs were the dominant population at time of stable disease, accounting for ~75% of the rare cell population, and only observable in low quantities (~10% of the rare cell population) at the time of progression (Figure 2H,I). Using targeted proteomics, we found that the morphometric features, such as CK SDOM, vimentin RFI and cellular eccentricity, differ between CTCs and CECs (Figure 2J,K), indicating that these parameters may differentiate between the cell state and cell type. Taken together, the three major rare cell types (EPI.CTC, pEMT.CTC and CEC) show unique morphometric and proteomic characteristics that differentiate them from CD45+ leukocytes (WBC) (Figure 2D).

### 3.2. Inter-Patient Assessment of Circulating Cell Types and Cell States in the Liquid Biopsy

**Figure 3:**
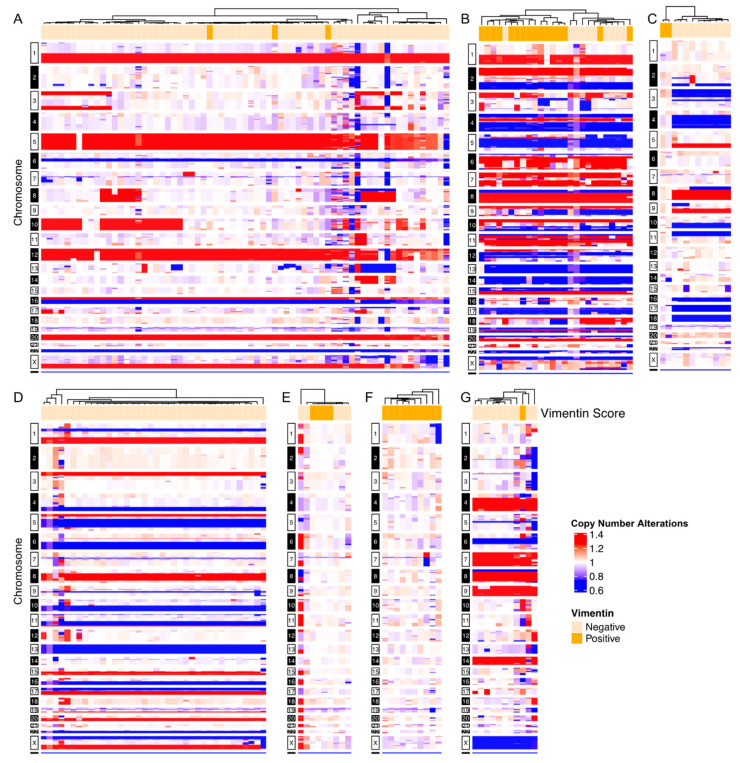
Representative single-cell whole-genome copy number alterations of breast and prostate cancer patients. CNA profiles are displayed as ratio to median. Vim status, as determined by immunofluorescence, is annotated on top of heatmaps, where Vim− cells = light orange and Vim+ cells = dark orange. Copy number gains are defined as >1.25 above median, losses as <0.75 below median and copy number neutral between 0.75–1.25. Copy number gains = red, copy number loss = blue, copy number neutral = white. (**A**–**F**) Single-cell CNV heatmaps of breast cancer patients (BC 1–6). (**G**) Single-cell CNV heatmap of a prostate cancer patient (PC 2).

To assess the generalizability of the observations made in the index patient, we followed the above experimental process for seven additional cases with confirmed CK+/CD45- cells, for which additional slides were available. A total of 321 CD45-/CK+ rare cells from six breast cancer patients and one additional prostate cancer patient were scored for vimentin expression and sequenced to determine genomic clonality [30,34]. Four cases had exclusively clonal cells (Figure 3A,B,D,G), two cases had a mix of clonal and non-altered cells (Figure 3C,E) and one contained exclusively non-altered cells (Figure 3F). Three of the seven cases consisted of clonal cells with variable vimentin IF expression, representing both pEMT.CTCs and EPI.CTCs (Figure 3A,B,G).

### 3.3. Targeted Proteomics Identifies Distinct Phenotypes

**Figure 4:**
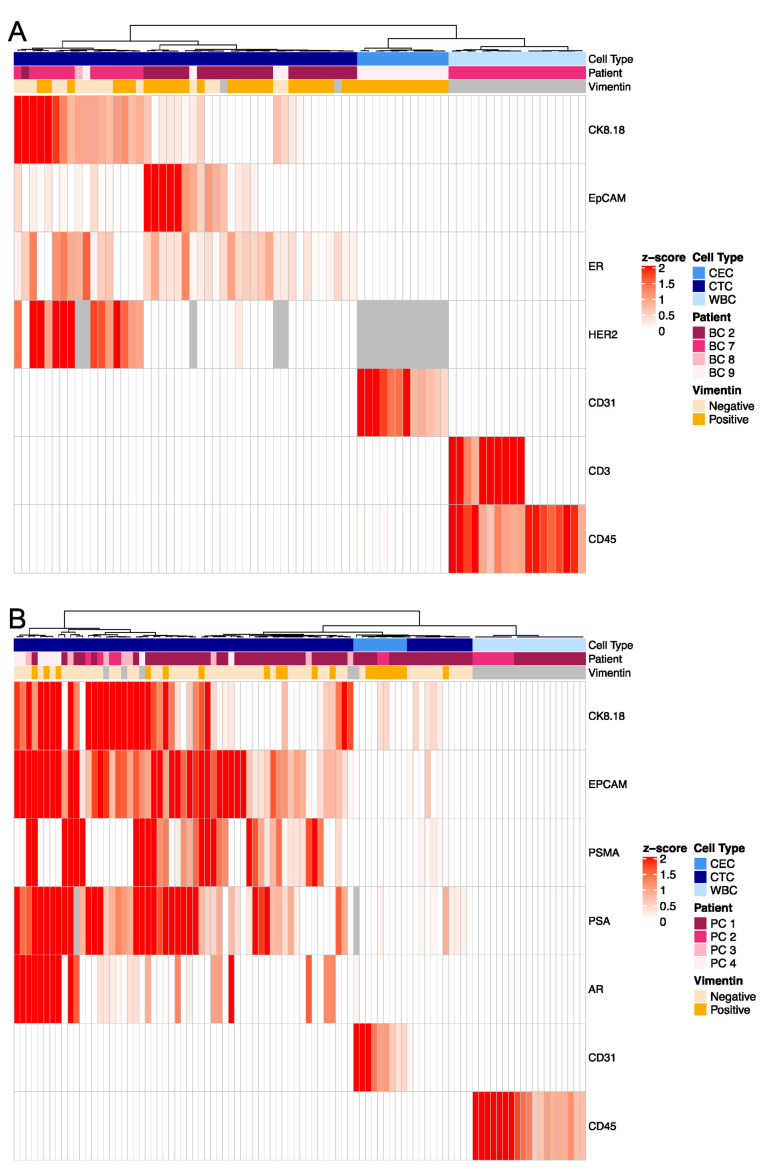
Multiplex proteomics of circulating rare cells and WBCs in patients with metastatic breast or prostate cancer. Multiplex proteomics of (**A**) breast cancer (BC 2, BC 7–9) and (**B**) prostate cancer (PC 1–4) patients. Grey = N/A; Not in panel.

The inter-patient characterization of CK+ rare cells was carried out in a similar manner to the index patient to further delineate specific cell types by targeted proteomics. We identified two major subgroups of rare cells, those expressing breast or prostate tissue markers such as the estrogen receptor (ER), human epidermal growth factor 2 (HER2), androgen receptor (AR), prostate-specific antigen (PSA) or prostate-specific membrane antigen (PSMA), and those that expressed the endothelial marker CD31 (Figure 4). Cells of both subgroups were negative for leukocyte-specific markers CD45 (BC and PC, Figure 4A,B) and CD3 (BC only, Figure 4A). This is consistent with the index patient data, in which two distinct cell types are present: epithelial (CTC) and endothelial (CEC) lineages. We noted high intra- and inter-patient heterogeneity of cancer specific markers on the CTCs.

### 3.4. Morphometrics and Multi-Omics to Separate Cell Types

**Figure 5:**
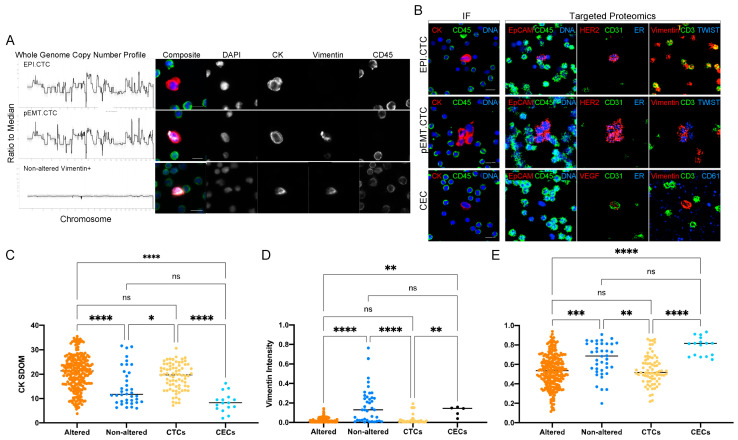
Morphometrics and multi-omics (**A**) CNA profiles together with IF images of representative EPI.CTC, pEMT.CTC and non-altered Vim+ cells from breast cancer patients BC 1–6. (**B**) Protein expression of representative EPI.CTC, pEMT.CTC and CEC by IF and targeted proteomics from patient BC 2. (**C**) CK SDOM, (**D**) Vim intensity and (**E**) cellular eccentricity of rare cells from all breast and prostate cancer patients (BC 1–9 and PC 1–4) assessed by CNV and IMC. Eccentricity is determined on a scale of 0–1, where 0 = circle and 1 = ellipse. The Kruskal–Wallis test with Dunn’s correction for multiple comparisons was used to test for differences between each group. *p*-value annotations: 0.1234 (ns), 0.0332 (*), 0.0021 (**), 0.0002 (***), <0.00001 (****). Scale bars are set to 10 μm.

Single-cell genomics was used to assess the copy number profile of rare cells detected and separate them into altered vs. non-altered groups. Figure 5A shows representative examples of Vim− and Vim+ clonal CTCs as well as Vim+ copy number-neutral rare cells from breast cancer patients. The protein expression of a representative EPI.CTC, pEMT.CTC and CEC derived from a breast cancer patient are shown in Figure 5B. Targeted proteomics distinguished CTCs and CECs based on their protein expression as described above. Due to the destructive nature in both the single cell genomics and the targeted proteomics, we assessed the morphometric features of rare cells identified by the IF assay that underwent subsequent characterization. Clonal cells showed a significantly higher CK expression as measured by the SDOM of the fluorescent intensity and were rounder compared to non-altered cells (Figure 5C,E). Similarly, in samples from prostate cancer patients, CTCs, as defined by the expression of a prostate-specific marker in proteomics, had a higher CK expression and presented a rounder phenotype compared to CECs. Vimentin expression was overall higher in non-altered cells and CECs, as defined by copy number analysis and multiplex proteomics, respectively (Figure 5D), compared to copy number altered cells and CTCs. Morphometrics and IF protein expression suggests that the clonal cells are the same cell type as the cells labeled as CTCs by multiplex proteomics. Similarly, morphometric parameters of the copy number-neutral cells resembled those labeled CECs by targeted proteomics.

### 3.5. Characterization of CECs

To further characterize the CEC cell type, we compared the CK and vimentin signals from the genomically altered CTCs and non-altered endothelial-like cells in the liquid biopsies of the cancer patients described above, to the endothelial cell lines (HPAECs and HUVEC) and to endothelial cells previously identified in patients with myocardial infarction (MI) [20].

**Figure 6:**
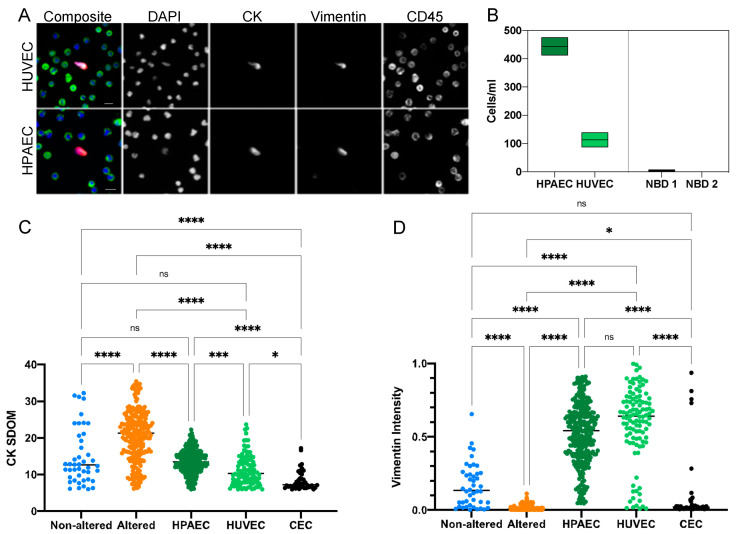
Comparison of endothelial cell lines with cells detected in MI and cancer patient blood samples. (**A**) Representative immunofluorescent images of HPAEC and HUVEC cell-line cells spiked into a NBD. (**B**) Rare cell enumeration of HPAECs and HUVECs compared to rare cells detected in NBD controls. HPAECs were spiked at approximately 430 cells/mL and had a recovery of 103%. HUVECs were spiked at approximately 100 cell/mL and had a recovery of 113%. (**C**) CK SDOM and (**D**) Vim signal intensity of spiked HPAECs and HUVECs cell line cells, CECs detected in MI patients, and CTCs and CECs detected in cancer patients. The Kruskal–Wallis test with Dunn’s correction for multiple comparisons was used to test for differences between each group. *p*-value annotations: 0.1234 (ns), 0.0332 (*), 0.0002 (***), <0.00001 (****). Scale bars are 10 μm.

Cultured HPAECs and HUVECs were spiked into the blood of NBDs at 400 and 100 cells/mL, respectively, in order to determine the detection efficiency of CECs after blood processing and plating on slides for the HDSCA workflow. Representative immunofluorescence images of the spiked cells stained for CK, vimentin and CD45 are shown in Figure 6A. Recovery was at or near 100% in each case (Figure 6B) showing that they can survive the processing with little or no loss.

The CECs from the MI patients (n = 3) were characterized in the original study as positive for DAPI, CD146 and von Willebrand Factor and negative for CD45 [18]. In this study, using antibodies against CK, vimentin and CD45 in rare cell analysis identified an average of 44 cells/mL (range 24.4–68.1, median 39.4 cells/mL) identified as CK+ Vim+ and CD45-.

When assessing the CK expression of the various endothelial cell populations identified above, we found substantial variation in the CK expression across the endothelial cell groups (non-altered cells in cancer patient samples, HPAEC, HUVEC and MI CECs); yet, all had significantly lower CK expression compared to the altered cells from the cancer patient group (Figure 6C). Conversely, the vimentin intensity detected in the CECs was highest in the HPAECs and HUVECs and lowest in the altered cell population detected in the cancer patient samples (Figure 6D). The MI CECs exhibited a range of vimentin expression comparable to the non-altered cells found in cancer patients.

## 4. Discussion

Liquid biopsy has the potential to provide significant new insights into tumor biology and cancer treatment at the individual patient level. However, to realize this potential it is necessary to develop the means to assay all the components in the liquid phase of the disease. Our methodology (HDSCA) examines all nucleated cells in the blood, without prior selection, and, thus, allows the use of multiple markers to detect an array of cell types. The results described here started as an effort to use new combinations of phenotypic and genomic markers to explore the unexpected cell types first observed in a longitudinal study of the index prostate cancer patient (PC1) [30]. We then extended this to test the generalizability of the results across additional breast and prostate cancer cases.

Our earlier results on the prostate cancer index patient (PC 1) showed that single-cell copy-number profiling of rare CK+ cells could distinguish clonally altered tumor cells (CTCs) from cells of an uncertain origin displaying neutral genomes, and, further, that both populations varied according to the state of the disease [40,41,42,43,44]. The data presented here take this observation further and demonstrate that through a combination of epithelial, mesenchymal, endothelial, and leukocyte markers, it is possible to further discriminate multiple cell types within the population of rare CK+ circulating cells. We show that we can detect CTCs, pEMT.CTCs, and CECs at varying frequencies in cancer patients using a non-enrichment-based detection platform.

In readdressing blood draws from patient PC1, using a combination of WGS, morphometrics and targeted proteomics, we found that copy number-neutral CK+ cells were morphologically distinct from clonal CTCs, had a high expression of the endothelial marker CD31, indicating an endothelial cell lineage (Figure 4 and Figure 5) and, further, trended toward a lower CK signal (CKdim) than the clonal cells. Endothelial cells, despite their mesodermal origin, have been reported to express certain CKs such as CK7, CK1, CK8 and CK18, explaining why they can be found using a pan-CK-based rare-cell detection assay [40,41,42,43,44]. Consistent with that result, we have shown that both spiked endothelial cell lines as well as MI CECs were robustly detected using CK and Vim as inclusion and CD45 as exclusion markers. While CTCs were more prevalent at times of disease progression in our index patient, we found high levels of CECs at the time of therapy response (Figure 2C,D,H). Although the reason for the increased presence of CECs at time of remission is unknown, it warrants further research.

Vimentin expression was detected not only in the majority of CKdim copy number neutral cells, as would be expected for endothelial cells, but also within the clonal population. Clonal Vim+ cells were morphologically similar to clonal Vim− cells and exhibited identical breakpoints in their copy number profiles, from which we surmise that these cells represent a subgroup of CTCs that has undergone at least a partial EMT conversion (Figure 2 and Figure 3).

Using targeted multiplexed proteomics, we detected varying expression levels of the tissue of origin markers such as ER and HER2 in the breast cancer cases, or AR, PSA, and PSMA in prostate cancer cases. This inter- and intra-patient heterogeneity of cancer-specific biomarkers is critical to note as there is an increasing awareness of how the low or heterogeneous expression of such markers (i.e., HER2) impacts the efficacy of targeted therapies [45]. In addition, we found that only approximately 50% of CTCs expressing the cancer-specific tissue of origin markers co-expressed EpCAM as assayed by IMC. This supports the observations by others that a dependence on EpCAM will only have limited sensitivity for the detection of CTCs [46,47,48,49]. In a similar manner, vimentin expression alone is insufficient to serve as the solitary marker for EMT, given that vimentin positivity is found in a range of cell types including cells of the tumor microenvironment, and immune cells, as well as CTCs. Yet, when combined with additional markers or analytical tools such as genomics or morphometrics, both EpCAM and vimentin can aid the classification and characterization of circulating rare cell types and states.

While our results show that we can reliably detect CECs with our assay, we recommend including known endothelial markers, such as CD31, vWF or CD146, for studies dedicated to CEC detection. To date, CEC detection is commonly performed by flow cytometry or a modified CTC detection platform; yet, there are few dedicated assays to detect CECs [18,22,50,51]. Given the differences in and the lack of standardization of CEC detection assays, as well as differences in enrolled patient populations, more studies are required to fully understand the role of CECs and their potential as a predictive biomarker. The same holds true for the characterization of pEMT.CTCs. Given the gradual and incomplete transition from an epithelial to a partial mesenchymal phenotype, multi-marker analysis by either multiplex proteomics or gene expression analysis is essential to correctly classify these CTCs. Yet, the combination of CK, vimentin and CD45 allows for the differentiation of circulating rare cells from leukocytes as a first pass, while ensuring to not omit CTCs with downregulated EpCAM expression (Figure 4). Downstream morphometric, genomic and proteomic assessment can then classify CRCs based on their cell type and state.

Our findings highlight the importance and feasibility of investigating not only cancerous cells, but also those of the tumor microenvironment from the liquid biopsy. It is to be expected that a comprehensive characterization of CECs and other cells present in the tumor microenvironment in conjunction with CTCs will lead to the improved means to measure the therapeutic response. Together, these results suggest that a multi-omic and multi-analyte analysis of the tumor and its microenvironment will be the future of precision oncology. Combined with longitudinal sampling enabled by liquid biopsy technologies, we have now the opportunity to trace the emergence of new tumor subclones together with circulating cells from the non-hematopoietic microenvironment at a single cell level using minimally invasive technology.

## 5. Conclusions

Our study highlights the importance of the multi-omic characterization of circulating rare cells and highlights the opportunity of detecting different cell types and cell states within one platform. While CK and vimentin expression alone cannot fully distinguish the cell types and cell states, additional parameters, such as morphology, genomic and multiplex proteomic analysis, aid to provide confidence in the classification of these cells. Enumeration of pEMT.CTCs could provide important insight into the aggressiveness of the disease and an opportunity for specific treatment selection. We propose that in the absence of CTCs, such as during disease remission, CECs could provide valuable information about the current disease state. In future studies, CECs might also give insight into metastatic mechanisms and disease progression through the longitudinal liquid biopsy analysis. While our results show the feasibility of detecting CTCs, pEMT.CTCs and CECs within one assay and associating them with disease states, more research is needed to fully elucidate the role of CECs in carcinomas. Lastly, although we highlight here specifically CECs, additional detection markers combined with morphometrics and multiplex proteomics have the potential to subclassify numerous more circulating TME subtypes and to elucidate their importance in tumor evolution, drug response and the seeding of metastasis.

## Figures and Tables

**Table 1:** Overview of prostate cancer patient stage and study IRBs.

Patient ID	Cancer Type	Stage	IRB #
PC 1	Prostate	Metastatic	UP-16-00691
PC 2	Prostate	Metastatic	UP-16-00643
PC 3	Prostate	Metastatic	UP-16-00691
PC 4	Prostate	Metastatic	UP-16-00691

**Table 2:** Overview of breast cancer patient subtypes, stage and study IRBs.

Patient ID	Cancer Type	Stage	Cancer Subtype	IRB #
BC 1	Breast	Metastatic	ER+/HER2−	UP-14-00592
BC 2	Breast	Metastatic	Triple Negative	UP-16-0070
BC 3	Breast	Metastatic	Triple Negative	UP-16-0070
BC 4	Breast	Metastatic	ER+/HER2−	UP-17-00882
BC 5	Breast	Metastatic	Triple Negative	UP-16-0070
BC 6	Breast	Metastatic	Triple Negative	UP-16-0070
BC 7	Breast	Metastatic	ER+/HER2−	UP-14-00182
BC 8	Breast	Metastatic	ER+/HER2−	UP-14-00523
BC 9	Breast	Metastatic	ER+/HER2−	UP-14-00523

**Table 3:** Overview of myocardial infarction (MI) patients.

Patient ID	Disease Type	IRB #
MI 1	MI	IRB-09-5287
MI 2	MI	IRB-09-5287
MI 3	MI	IRB-09-5287

**Table 4:** Antibodies used for targeted proteomics by imaging mass cytometry.

Metal Tag	Target	Antibody Clone	Final Dilution
Yb174	CK8/18	C51	1:50
Pr141	EpCAM	9C4	1:100
Dy163	ER	D8H8	1:100
Tb174	HER2	42c/erbB2-2	1:100
Nd148	HER2	29D8	1:100
Y89	CD45	HI30	1:200
Tm169	PSMA	460420	1:100
Gd160	PSA	TD11B3-4	1:100
Sm154	AR	G122-434	1:100
Ir193	DNA		
In115	Membrane		

## Data Availability

The data presented in this study are openly available in the BloodPAC Data Commons at https://data.bloodpac.org/discovery/BPDC000131, reference number BPDC000131.
